# Motor and Cognitive Trajectories in Infants and Toddlers with and at Risk of Cerebral Palsy Following a Community-Based Intervention

**DOI:** 10.3390/children11111283

**Published:** 2024-10-24

**Authors:** Kanishka Baduni, Allison McIntyre, Caitlin P. Kjeldsen, Larken R. Marra, William C. Kjeldsen, Melissa M. Murphy, Owais A. Khan, Zhulin He, Kristin Limpose, Nathalie L. Maitre

**Affiliations:** 1 Department of Kinesiology, University of Georgia, Athens, GA 30602, USA; kanishka.baduni@uga.edu (K.B.); owais.khan@uga.edu (O.A.K.); 2 Department of Pediatrics, Emory University School of Medicine, Atlanta, GA 30322, USA; allison.mcintyre@emory.edu (A.M.); caitlin.kjeldsen@emory.edu (C.P.K.); larken.marra@emory.edu (L.R.M.); william.chase.kjeldsen@emory.edu (W.C.K.); melissa.murphy@emory.edu (M.M.M.); zhulin.he@emory.edu (Z.H.); klimpos@emory.edu (K.L.); 3 Children’s Healthcare of Atlanta, Atlanta, GA 30322, USA

**Keywords:** cerebral palsy, motor development, cognition, early intervention

## Abstract

Background: Early motor development is fundamental in driving cognitive skill acquisition. Motor delays in children with cerebral palsy (CP) often limit exploratory behaviors, decreasing opportunities or the quality of cognitive development, emphasizing the importance of early intervention. This study aimed to assess immediate and 5-month motor and cognitive changes in infants and toddlers at risk of or with CP after participation in a community-based program. Methods: Twenty-two children (mean age: 22 ± 7 months) classified using the Gross Motor Function Classification System (GMFCS) and mini-Manual Ability Classification System (mini-MACS) participated in a 6-day community-based activity program, with outcomes assessed using the Developmental Assessment of Young Children (DAYC-2). Results: Participants who met their motor goals post-participation had significantly higher cognitive scores (*p* = 0.006) 5 months after the program. Participants with higher functional motor abilities (GMFCS levels I–II, *p* = 0.052; mini-MACS levels I–II, *p* = 0.004) demonstrated better cognitive scores at 5 months, adjusted for baseline scores, than those with lower functional motor abilities. Conclusions: This study highlights the impact of motor improvements following an evidence-based community program on later cognitive development. Prospective studies investigating the mechanisms and mediation of cognitive progress in children with CP should investigate the effects of early motor interventions on long-term developmental trajectories.

## 1. Introduction

Motor development in infancy is foundational to cognitive, communicative, social, and emotional development, shaping how children engage with and understand the world around them [1,2,3]. Through motor exploratory activities, infants gather sensory information about objects, test their relationship with the environment, and probe interactions between their bodies, objects, and surroundings, all essential to the development of advanced cognitive abilities [4,5]. For children with motor disorders like cerebral palsy (CP) [6], impairments can limit the opportunity for and quality of exploratory behaviors [4]. As neuroplasticity peaks during the first three years after birth [7], early interventions targeting motor development have the potential to have the greatest effect on long-term developmental trajectories and have been the focus of seminal research [8,9,10,11,12]. Despite a substantial body of interventional studies, there remains a significant gap in research concerning how motor improvements achieved through early interventions influence cognitive development.

Developmental theories like Dynamic Systems Theory (DST) and embodied cognition emphasize the integral contribution of motor skills to cognitive development [13,14,15], and are supported by empirical evidence from neuroimaging [16,17,18] and behavioral studies [19]. The DST views development as a non-linear interaction involving the individual, task, and environment, while embodied cognition asserts that cognitive processes are deeply rooted in physical actions. Neural models like Dynamic Field Theory highlight stable neural activation patterns across sensorimotor brain areas during motor skill acquisition, and suggest a bidirectional relationship where movement fuels cognition, and cognition propels movement [20]. These interactions enhance neural plasticity, with sensorimotor experiences shaping neural pathways and creating cascading effects that enhance both motor and cognitive functions. Early motor impairments can lead to developmental challenges that affect cognitive abilities, making it crucial to identify early motor–cognitive links in children born preterm or with CP to better understand developmental trajectories in these vulnerable groups [21].

To better understand this motor–cognition link, it is essential to categorize motor skills into gross motor skills, involving large muscle groups for actions like walking and jumping, and fine motor skills, which require dexterity for tasks like picking up small objects. Gross and fine motor skills, including manual abilities, are associated with cognitive development in early infancy [22,23,24,25,26,27,28]. Manual abilities encompass dexterity-based tasks that require coordination, precision, and the integration of both gross and fine motor skills [29]. Empirical evidence also suggests that while motor skills support cognitive advancement, cognition reciprocally scaffolds motor mastery [13,15,20,30]. Various aspects of cognition can be tested from executive function to fluid and crystallized intelligence; however, the most common aspect clinically tested in the under-three population is general cognition, encompassing decision-making abilities, functional memory use, play imitation, and purposive planning [29,31,32].

Child development involves a dynamic and interconnected relationship between motor and cognitive skills. Research consistently demonstrates that goal-directed intervention strategies promote motor development in early childhood [10,33]. Specifically, community-based, child-led, and goal-focused interventions and programs have been shown to significantly improve motor and functional outcomes. This approach aligns with the International Classification of Function and Health in Disability (ICF) [34] and its translation into practice using the F-words framework, which emphasizes six key areas: function, family, fitness, fun, friends, and future, all of which prioritize what is meaningful to the child and family [35]. Empowering caregivers with education and support systems not only promotes ongoing developmental progress but also strengthens the caregiver–child bond, which is vital for the child’s overall development [36,37]. As such, the state of Georgia helped fund an evidence-based week-long summer program for families of children at risk of or with CP. In this program, children participated in motor-focused, child-directed, goal-oriented, activities, while family social dynamics and participation were encouraged through a caregiver–child-focused workshop aimed at empowering families. This community program (Climbing Activities Music and Parents, CAMP) also represented the implementation into practice of published clinical principles shown to improve outcomes in this population. By facilitating motor achievements and supporting caregivers, the community CAMP enabled children with CP to reach motor goals while enhancing their parents’ self-efficacy.

This study explored how motor goal attainment may contribute to cognitive development by investigating whether achieving motor goals post-CAMP leads to long-term improvements in cognitive milestones. Using a prospective observational design, we hypothesized that children who achieved their motor goals immediately post-CAMP would have significantly higher cognitive scores at the five-month follow-up. Additionally, we examined how gross motor function and manual ability influenced both immediate post-CAMP goal achievement and five-month motor and cognitive outcomes.

## 2. Materials and Methods

### 2.1. Participants

We used a convenience sampling method for participant recruitment. We organized a community activity designed for families of infants and toddlers at risk of or diagnosed with CP. Recruitment spanned the state of Georgia (USA), utilizing various channels such as referrals from ongoing lab studies, Facebook posts, flyers, and direct communication in high-risk infant follow-up programs or community early intervention providers. The activity was available to all families without cost due to state and donor funding, and participation in the associated research study was optional. All families attending CAMP consented to also participate in an observational research study with follow-up, approved by the Institutional Review Board at Emory University (ID—STUDY00006010 and STUDY00006666).

Inclusion criteria comprised children aged 9–36 months (corrected age for those born preterm) with an interim clinical diagnosis of being at high risk of CP or a confirmed CP diagnosis based on established guidelines [38,39]. The two participants designated as high risk for CP received a confirmed CP diagnosis within six months after the conclusion of CAMP. Additionally, caregivers needed to be fluent in English or have access to an interpreter for CAMP activities.

### 2.2. CAMP Setting

The CAMP program took place in the Baby Brain Optimization Project building in Atlanta, Georgia for three alternating weeks between June and July 2023, with 6–8 families per week. Activities were co-led by a transdisciplinary team, comprising licensed physical, occupational, speech, and music therapists, as well as undergraduate and graduate students from universities across Georgia. Concurrent parent workshops, integral to the program, were developed and conducted by psychologists certified in the Triple P (Positive Parenting Program) [40], social workers, health educators, and case managers. Baseline comprehensive evaluation of CP characteristics, medical and behavioral complexity, and social determinants of health were also performed by the medical team.

In addition to 15–20 min of structured dyadic activities focused on multisensory communication and socialization at the opening and closing of each day, infants and toddlers engaged in two hours of activity across 4 stations with supported breaks to account for child endurance and necessary functions [41]. Participants were paired into groups of two by the therapy team following pre-CAMP assessments, based on similar goals and temperaments [42]. Each station’s workflow aligned to meet initial goals (Table 1).

Goal setting: Caregivers were asked to set a functional, family-centered goal with the CAMP medical team during an initial goal setting session. This included a short- (CAMP) and long-term (4-month) goal in a collaborative process aimed to establish a family-oriented community goal over four months tailored to each family’s needs, aspirations, and the ways they could enhance participation in family life. If the caregiver had difficulty identifying functional goals, then the medical team assisted them through motivational interviewing to identify family-centered goals. This was recorded as the family GAS (Goal Attainment Scale) goal and shared with the CAMP staff during a multidisciplinary team meeting; the 4-month GAS goal was combined with the baseline assessment to establish a measurable post-CAMP goal. While CAMP goals comprise various aspects of the ICF, only motor goals are considered in the current report.

1. A gross motor station featured a custom gravity-assisted support system (Enliten LLC, Newark, NJ, USA) to aid in mobility skill development by decreasing the effort necessary for anti-gravity skills and decreasing the pressure felt during falls or stumbles [43,44]. Pressure support started at 30% of body weight and was rapidly decreased until weight-bearing and muscle activation efforts were visible. Music therapy was provided with physical therapy to facilitate understanding of rhythmic patterns during ambulatory activities [45,46]. Play-based obstacle courses encouraged postural transition skills and gait adaptations for improving gait stability.
2. A climbing station targeted coordination, selective motor control, motor planning, and visual motor skills. Activities utilized a toddler-sized rock-climbing wall and modular climbing structures to enhance vestibular, gross motor, and reaching skills [47,48,49]. The station was co-led by an occupational therapist and an adaptive climbing athlete.
3. A balance station addressed core strengthening and postural stability. The station was led by music therapists with the assistance of physical therapists to design and modify activities. The station incorporated music therapy and toddler-adapted yoga for attunement, socialization, and attention [50,51]. Balance challenges increased with progressive opportunities to experience disequilibrium as trunk control increased.
4. A picnic and play station focused on fostering communication, manual abilities, social skills, and self-feeding. The station was designed and led by speech therapists and occupational therapists. The station incorporated snack time and play skills, with goals based on the child’s skills and needs.

Circle time: Caregivers participated with their children in a circle time session at the start and end of each CAMP half-day, facilitated by a music therapist. This time was dedicated to music-based caregiver–child engagement, fostering opportunities for bonding, and creating a sense of connection amongst dyads and across the group [52].

Caregiver workshops: While children participated in “station” activities, the caregivers attended a series of support workshops covering how to access caregiver resources in Georgia, how to access therapy services, positive parenting techniques tailored to infants and toddlers with CP, practical tools for promoting mental health and self-care, navigating their child’s healthcare journey, and understanding the ICF model [34]. These efforts supported caregiver involvement and parent-to-parent connection, which has been shown to enhance the success of early interventions [33]. The rationale for incorporating caregiver activities was based on evidence [36] highlighting the need for caregiver education, access to resources, and peer support. Not only does effective caregiver support help build a strong caregiver–child relationship, essential for child development [37], it increases the likelihood that caregivers will engage in positive parenting practices that enhance developmental outcomes [53]. Family GAS goals were incorporated into the workshops to empower caregivers with specificity. On the last day, caregivers received hands-on instruction and coaching from CAMP staff to support their child’s motor goals, along with personalized plans called roadmaps, outlining actionable steps for short-term goal achievement post-CAMP and a customized healthcare roadmap for the year ahead [54,55].

### 2.3. Measures

Baseline measures, family GAS goals, and CAMP goals were collected on the day before the start of the CAMP activities. Outcomes were measured after 5 days of CAMP. A telehealth follow-up was conducted with families five months later with standardized measures (described below) and a scripted interview aimed at assessing the CAMP experience, feasibility, and acceptability.

Motor and cognitive milestone acquisition was evaluated using the Developmental Assessment of Young Children-2 (DAYC-2) [56] and the cognition and physical development domains via telehealth. The DAYC-2’s suitability for telehealth follow-up was demonstrated during the COVID-19 pandemic [57], as it integrates both caregiver interviews and direct child observation. Due to the brief 6-day interval between baseline and post-CAMP evaluations, raw DAYC-2 scores were used for all analyses rather than norm-referenced scores. The administration of the DAYC-2 was standardized by adapting all questions into lay terms where necessary and ensuring that ceiling-level items were tested according to the standard procedures outlined in the DAYC-2 manual. A video workshop created by the DAYC-2 gold-standard examiner demonstrated proper administration techniques. This was followed by scoring practice with video exams, where all examiners practiced until they achieved over 90% reliability, as verified by the gold-standard examiner. Telehealth administration followed a modified written protocol [57] and was conducted by two DAYC examiners (KB, WK) who had already achieved the required in-person reliability.

The Gross Motor Function Classification System (GMFCS) [58] and mini-Manual Ability Classification System (mini-MACS) [59] were used to classify participants’ motor and manual abilities, respectively. These classifications help in understanding the functional abilities of children with cerebral palsy across different levels. Three experienced therapists jointly assessed and classified all participants after the baseline evaluation to ensure consistent and accurate classification using both GMFCS and mini-MACS levels

Gross motor station goal achievement at the end of CAMP, referred to as post-CAMP motor goals, were recorded as binary data (1 for achievement, 0 for non-achievement). Additionally, the Goal Attainment Scale (GAS) [60] was used to monitor individual short-term goals, termed family GAS goals, targeted for achievement within three to four months. The GAS serves as an individualized metric to quantify progress toward personalized goals, utilizing an ordinal scale that typically ranges from −2 to +2, thereby providing a nuanced measure of goal attainment.

### 2.4. Statistical Analysis

This observational study compared two groups: those who achieved their motor goal post-CAMP and those who did not. Generalized Linear Mixed Models (GLMMs) were utilized, adjusting for corrected age, with participant ID included as a random effect to account for intra-individual variability. Main effects were further explored using post-hoc within- and between-group comparisons, with effect sizes reported as Cohen’s d [61]. A power analysis for the GLMM, accounting for the correlation of repeated measures, was conducted using G*Power version 3.1.9.6 [62]. With α set to 0.05 and assuming a medium correlation (0.5) and medium effect size ($f^{2}$ = 0.33) based on previous literature [25,63,64,65], a post hoc power analysis determined that a sample size of 22 provided 80% power to assess the primary hypothesis of whether post-CAMP goal achievement led to long-term cognitive milestone improvement. These findings are considered robust within the study parameters. Baseline characteristics and GAS goals were compared between the two groups using Wilcoxon rank-sum tests for continuous variables and Fisher’s exact tests for categorical variables. Links between motor and cognitive outcomes were assessed using Spearman rank correlation.

## 3. Results

Our study cohort included 22 infants and toddlers (mean age = 22 ± 7 months) from a diverse community of children with CP in the state of Georgia (Table 2). The follow-up rate at five months was 100%. At the post-CAMP assessment, 64% of participants met their post-CAMP motor goals, and 86% achieved their four-month family GAS goals. Baseline assessments showed differences in GMFCS and mini-MACS levels between groups who attained their post-CAMP goals and those who did not (Table 3). Developmental raw scores over time are presented in Table 4. At the five-month follow-up, 91% of families rated the CAMP goal-setting workshop as extremely or very helpful, highlighting its positive impact on supporting their children’s developmental goals.

### 3.1. Post-CAMP Motor Goal Success and Long-Term Cognitive Gains

**Figure 1:**
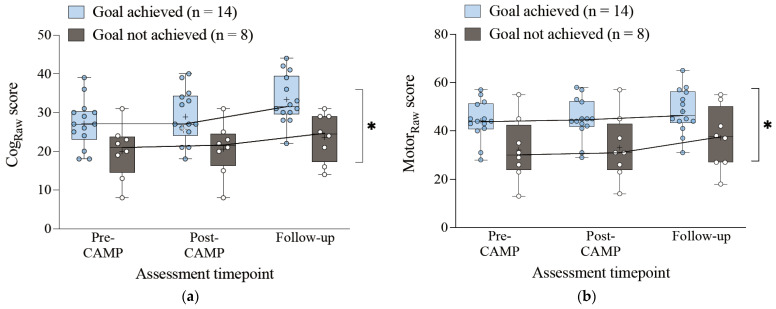
Changes in DAYC-2 raw scores across assessment timepoints, not controlled for age, for (**a**) cognitive score and (**b**) motor scores (means are indicated by +). * Statistically Significant differences between groups across all time-points.

After adjusting for pre-CAMP scores and corrected age in the GLMM, cognitive score changes were higher at the five-month follow-up for participants who met their post-CAMP goals than for those who did not (β = 9.4, *p* = 0.006) (Table 5). A similar pattern was observed for motor scores (β = 11.9, *p* = 0.029). Post-hoc analyses revealed statistically significant differences between baseline and post-CAMP scores for those who achieved their motor goals (cognitive: *p* = 0.004; motor: *p* = 0.007), but not for those who did not achieve their motor goals (cognitive: *p* = 0.102; motor: *p* = 0.059). Statistically significant differences between post-CAMP and five-month follow-up scores were observed for those who achieved their CAMP goals, with moderate to large within-group effect sizes (cognitive: d = 0.67, *p* = 0.001; motor: d = 0.37, *p* = 0.008). For those who did not achieve their motor goals, a statistically significant improvement was only observed in the motor domain at follow-up, with moderate effect sizes (cognitive: d = 0.46, *p* = 0.092; motor: d = 0.31, *p* = 0.035). Notably, even participants who did not achieve their post-CAMP goals displayed a positive trend in cognitive and motor skill progression at follow-up (Figure 1). Comparing family GAS goal achievement at 5 months between those who achieved their post-CAMP motor goals (100%) and those who did not (63%) did not show statistical significance (*p* = 0.059).

### 3.2. Motor Function Abilities Influencing Cognitive and Motor Outcomes

Preliminary analysis solidified the significant positive correlations between motor function classification (GMFCS, *r* = 0.48, *p* = 0.012; mini-MACS, *r* = 0.53, *p* = 0.006) and cognitive scores at baseline. Additionally, we found that GMFCS and mini-MACS levels differed significantly at baseline between participants who achieved their post-CAMP goals and those who did not. Participants with higher gross and manual abilities were more likely to achieve their post-CAMP goals, with 91% of those successful classified as level I (Table 3).

We conducted further exploratory analyses examining how GMFCS and mini-MACS levels were associated with cognitive and motor outcomes both immediately post-CAMP and at follow-up (Table 6). In the gross motor model, age-corrected cognitive scores in those at GMFCS level III trended lower than those at GMFCS level I–II (*p* = 0.052). In the manual ability model, cognitive scores in those at level III were significantly lower (*p* = 0.004). In both models, participants at levels IV–V had significantly lower cognitive scores compared to the reference group, suggesting that greater motor and manual impairments were linked to poorer cognitive outcomes. Time was also a significant factor, with follow-up scores showing greater improvements in both cognitive and motor outcomes (*p* < 0.001). Confidence intervals for immediate post-CAMP outcomes included zero, suggesting variability and uncertainty in short-term results (Table 6).

## 4. Discussion

Our primary aim was to assess whether achieving short-term motor goals resulted in longer-term cognitive gains in children at risk of or diagnosed with CP. Our results showed that participants who met their motor goals after a one-week community-based program displayed greater improvements in cognitive scores at the five-month follow-up than those who did not. This finding, in children aged three and under with motor impairments characteristic of CP, supports existing research that associates motor gains with cognitive development in this population [21]. It further supports the importance of addressing motor skills during the first three years, as small early motor gains may contribute to larger cognitive and motor improvements in the following months. This is particularly significant since these gains are driven by family-set goals.

Cognitive development in early childhood depends on sensorimotor exploration, where movement refines cognitive processes through sensory feedback and motor planning [5,15,21,32,66]. Sensorimotor experiences play a key role in establishing foundational cognitive skills, such as executive function, which supports higher-level processes like problem solving and planning [16,67,68]. Neuroimaging studies reveal that motor and cognitive processes are interlinked, particularly involving the prefrontal cortex and cerebellum [16,17,18]. These findings emphasize neuroplasticity, where motor experiences during early development reorganize neural pathways and enhance cognitive skills. The disruption of sensorimotor pathways following early brain injury in CP may hinder exploratory behaviors and affect cognitive development [21,69,70]. Early motor gains have been associated with cognitive skill enhancement in children with CP [9]. In our study, the lack of immediate significant changes in cognitive scores after the short-term CAMP program may be attributed either to the brevity of the time interval or to the DAYC-2 assessment tool’s limited sensitivity to detect subtle cognitive changes in the short term [56]. However, effect sizes for within-group comparisons were consistently larger in the cognitive domain compared to motor score progression, indicating positive trends associated with participation in a community-based, child-led, sensorimotor play-based program.

Our analysis of cognitive outcomes across varying levels of gross motor function revealed positive but non-significant trends between GMFCS levels and cognitive scores (*p* = 0.052), possibly due to sample size and variability. However, when manual abilities were assessed using the mini-MACS, cognitive scores showed significant differences across grouped levels (*p* = 0.004), suggesting that manual abilities may be a more sensitive predictor of cognitive outcomes than gross motor function [71]. While some studies emphasize the importance of gross motor function in predicting cognitive development [19], it is important to note that hand strength, a key aspect of manual ability, was also assessed in their research. This aligns with literature suggesting that manual skills are particularly crucial for cognitive tasks, as many cognitive assessments rely on fine motor abilities for successful completion [26,72,73]. Our findings are consistent with multiple studies that emphasize a stronger link between manual abilities, fine motor skills [74], and cognitive development in early childhood [22,23,24,25]. Additionally, Cabral et al. [72] demonstrated that in children with CP, manual ability significantly influenced performance on the Bayley Scales of Infant and Toddler Development-III (Bayley-III) cognitive test, especially on tasks requiring fine motor skills. Given the similarities between the Bayley-III and the DAYC-2 scale used in our study [56], this connection may explain the association between manual abilities and cognitive outcomes in our findings.

The primary goal of this study was not to evaluate the effectiveness of the CAMP program, as the observational nature of the design prevents causal attribution. However, we can relate some findings to the context of the existing literature on structured, goal-oriented interventions in community settings for children with motor delays [35,75,76,77]. Following CAMP, 64% of participants met their post-CAMP motor goals. Various studies on short-term interventions have documented motor goal achievements using standardized tools [76,77,78]. For example, an 8-day manual skills-focused camp for school-aged children showed that over 80% of participants achieved their weekly motor goals [79]. This success underscores the dynamic nature of motor skill development and the importance of using longitudinal assessments to assess goal achievement [80,81]. Future prospective studies evaluating the effectiveness of the CAMP as an intervention will provide important information on causal attribution.

Longer periods allow for a more comprehensive understanding of developmental trajectories over time. The 86% success rate in meeting family GAS goals at follow-up highlights the importance of tracking long-term developmental progress and goal achievement within community-based programs. Notably, participants with lower motor function showed delayed but significant improvements in both cognitive and motor abilities at follow-up (Figure 1), and 64% of participants who did not achieve post-CAMP motor goals still met their family GAS goals. This may be attributed to the emphasis on the sustained involvement of empowered caregivers and the educational support provided to families during CAMP. These findings support research identifying community settings as valuable platforms for delivering interventions to children with CP [54,82]. The high rate of goal achievement reflects the effectiveness of these structured approaches, bolstered by caregiver education and peer-to-peer support during community camp. Caregivers reported that 90.9% found the goal-setting workshops helpful. The individualized roadmaps provided ongoing support, enabling families to continue developmental progress post-CAMP.

## 5. Limitations

In addition to the drawbacks of the assessment tools outlined above, this study had other limitations such as the small sample size reducing the precision and generalizability of the findings, which is reflected in the variability in our data. The absence of a control group further limits definitive conclusions. Our sample also had extreme variability across body function and environmental and personal factors, which was reflected in the results. We acknowledge that the intertwined nature of motor and cognitive development, alongside the multifaceted approach of the CAMP program, complicates pinpointing whether cognitive improvements stemmed from motor gains, enhanced parent–child interactions, or a combination of factors. Future research should explore these mediators to clarify their impact on development. However, this same variability can also present an opportunity. In CP, the relationship between motor and cognitive impairments is complex and non-linear, with only a subset of children experiencing severe cognitive difficulties [83]. This variability, compounded by environment and family factors, allows for the exploration of factors within the relationship between motor skill and cognition that may not be as evident in older children. By investigating early cognitive skill development and providing multisensory and community-based programs that target more than motor systems, this study sought to utilize developmental variability by leveraging a community setting where interventions can be tailored to individual needs.

Given the complex and non-linear nature of developmental trajectories in those with early brain injuries, adaptive methodologies and sensitive assessment tools are required to characterize these evolving trajectories. Future research should incorporate more granular and sensitive measures to track short-term changes in cognitive and motor domains. Adequately powered studies with larger samples and tightly matched controls are needed to isolate intervention effects and provide robust evidence of efficacy. Additionally, single-subject designs could offer deeper insights into individual responses to interventions. While this study employed an intense short-term program, which can be viewed as a limitation, future research should consider exploring longer intervention-based program durations or increased dosage to fully understand the long-term effects. This approach transforms potential limitations into valuable opportunities for refining targeted therapy and research, ultimately optimizing early interventions for individual developmental trajectories.

## 6. Conclusions

The current study focuses on the links between immediate motor skill improvements following a community-based program and subsequent cognitive progress in infants and toddlers at risk of or diagnosed with CP. The findings suggest that targeting motor abilities can contribute to cognitive gains through evidence-based, goal-directed, multisensory interventions across the ICF domains. While programs like CAMP that are community-based, family-centered, and child-driven in nature offer valuable insights into the motor–cognitive relationship, this study does not aim to guide practices based on the CAMP model as an intervention. Instead, it emphasizes the importance of understanding how motor function influences developmental trajectories, particularly in children with developmental delays. By assessing motor and cognitive outcomes over time, such programs allow researchers to explore how early motor improvements impact broader developmental pathways. This understanding is critical for informing future research on motor–cognitive relationships and developing more comprehensive support strategies for children with developmental delays.

## Figures and Tables

**Table 1:** Example of goal setting: breaking down community and family-centered GAS goals into specific post-CAMP goals for a participant.

Family GAS Goal	CAMP Goals
For my child to be able to walk around the house while holding my hand and to use gestures to express their needs during family meals, in a way that their siblings can understand too, within 4 months	**Gross Motor Station:** Child will be able to walk 15 feet with moderate assistance around the trunk and will maintain standing while taking support anteriorly from a mirror while playing with sticky toys within 5 days
	**Climbing Station:** The child will crawl over a small 15-inch-high tunnel with minimal assistance from the therapist within 5 days
	**Balance Station:** Child will be able to maintain balance while sitting, with minimal support at the trunk, on a dynamic surface, such as a medicine ball, moving side-to-side within 5 days
	**Picnic and Play:** Child will be able to play with the therapist and a peer while waiting for his turn and communicate “more” to the therapist through gestures or words within 5 days

**Table 2:** Demographics of Participants.

Characteristic	Frequency [N = 22] ^1^
Sex	
Females	9 (41%)
Males	13 (59%)
Maternal Education	
Less than 7th grade	1 (4.5%)
Partial college or trade school	3 (13.6%)
College graduation	8 (36.4%)
Graduate education	9 (40.9%)
Prefer to not answer	1 (4.5%)
Race	
Black/African American	10 (45.5%)
Caucasian	9 (40.9%)
Mixed	1 (4.5%)
Other/Prefer to not answer	2 (9.0%)

^1^ *n* (%).

**Table 3:** Characteristics of CAMP Groups.

Characteristic	Overall, N (22) ^1^	CAMP Goal Achieved, N (14) ^1^	CAMP Goal Not Achieved, N (8) ^1^	*p*-Value ^2^
CA ^3^	22 (7)	20 (5)	25 (9)	0.065
GMFCS				0.006 *
Levels I–II	11	10	1	
Level III	5	3	2	
Levels IV–V	6	1	5	
MACS				0.002 *
Levels I–II	12	11	1	
Level III	5	2	3	
Levels IV–V	5	1	4	

^1^ *n*; ^2^ Wilcoxon rank sum exact test; ^3^ corrected age (CA) reported in months; mean (SD); * statistically significant difference between CAMP goal achieved and goal not achieved groups.

**Table 4:** Cognitive and Motor DAYC raw scores between groups from baseline to 5 months.

	Pre-CAMP			Post-CAMP			Follow-Up		
	Overall	Goal Achieved	Goal Not Achieved	Overall	Goal Achieved	Goal Not Achieved	Overall	Goal Achieved	Goal Not Achieved
Cognition ^1^	24.55 (7.21)	27.14 (6.13)	20.00 (7.01)	25.91 (7.78)	28.93 (6.75)	20.63 (6.82)	29.82 (7.75)	33.36 (6.25)	23.63 (6.23)
*p*-value ^2^ (Cohen’s *d*)		0.036			0.013 (1.24)			0.003 (1.56)	
Motor ^1^	39.82 (11.54)	44.21 (8.12)	32.13 (13.07)	40.59 (11.73)	44.93 (8.36)	33.00 (13.38)	44.18 (11.69)	48.21 (9.10)	37.13 (12.91)
*p*-value ^2^ (Cohen’s *d*)		0.040			0.040 (1.15)			0.040 (1.05)	

^1^ Mean (SD); ^2^ between-group comparison (goal achieved vs. goal not achieved) using Wilcoxon rank sum exact test.

**Table 5:** GLMM for CAMP Goals Group.

Characteristic	Cognitive			Motor		
	Beta	95% CI	*p*-Value	Beta	95% CI	*p*-Value
Model 1:						
CAMP Goal Group *			0.006			0.029
Achieved	9.4	3.0, 15.7		11.9	1.4, 22.4	
Time			<0.001			<0.001
Post	1.4	0.0, 2.7		0.8	−0.5, 2.1	
Follow Up	5.3	4.0, 6.6		4.4	3.1, 5.7	

* Group—CAMP goal not achieved was the reference level while controlling for pre-CAMP scores.

**Table 6:** GLMM for GMFCS Groups, and Mini-MACS Groups.

Characteristic	Cognitive			Motor		
	Beta	95% CI		Beta	95% CI	
Model 2a:						
GMFCS Group *			0.052			<0.001
Group III	−4.7	−12.7, 3.3		−12.7	−21.8, −3.7	
Groups IV–V	−8.8	−17.2, −0.5		−21.9	−31.3, 12.5	
Time			<0.001			<0.001
Post	1.4	0.0, 2.7		0.8	−0.5, 2.1	
Follow Up	5.3	4.0, 6.6		4.4	3.1, 5.7	
Model 2b:						
Mini-MACS Group *			0.004			<0.001
Group III	−0.7	−7.5, −6.0		−10.2	−17.9, −2.6	
Groups IV–V	−12.2	−18.8, −5.3		−23.9	−31.6, −16.3	
Time			<0.001			<0.001
Post	1.4	0.0, 2.7		0.8	−0.5, 2.1	
Follow Up	5.3	4.0, 6.6		4.4	3.1, 5.7	

* Groups I–II were the reference levels while controlling for pre-CAMP scores.

## Data Availability

The original contributions presented in the study are included in the article, further inquiries can be directed to the corresponding author.
